# Exploring the impact of case mix factors on length of stay to a Complex Discharge Unit across three phases: pre-COVID-19 (2019), during COVID-19 (2021) and late-stage COVID-19 (2023) pandemic

**DOI:** 10.1007/s11845-025-04072-6

**Published:** 2025-09-03

**Authors:** Tahira Bibi, Mbuotidem Udongwo, Joseph Deegan, Tom Cuddihy, Alanna Crawford, David Griffin, Keneilwe Malomo, Mohammed Tariq Zainal, Patrick Mc Cluskey, Haswadi Hassan, Declan Byrne, Joseph Browne, Ontefetse Ntlholang

**Affiliations:** 1 Acute and General Medicine Department, St James’s Hospital, Dublin 8, Ireland; 2 Geriatric Department, St Mary’s and Mater Misericordiae University Hospital, Dublin 7, Ireland; 3 Discipline of Medical Gerontology, Trinity College Dublin, Dublin 2, Ireland

**Keywords:** Complex Discharge Unit, COVID-19, Length of stay, Medicolegal issues, Multimorbidity, Pre-COVID-19 and late stage-COVID-19

## Abstract

**Background/aims:**

Subacute complex discharge units (CDUs) offer intermediary person-centred care between acute hospital and community services by providing specialised care for patients with complex medical and functional needs. However, several elements of clinical practice were affected during the COVID-19 pandemic. We aimed to determine the impact of several case mix factors on length of stay and how this impact changed across three phases: pre-COVID-19 (2019), during COVID-19 (2021) and late-stage COVID-19 (2023) in our Complex Discharge Unit.

**Materials and methods:**

Before collecting data, our institution’s Research and Innovation Office approved the study (reference number: 8900, on the 23rd of May 2024). All patients (920) who were admitted to our 23-bed Complex Discharge Unit in 2019 (320 patients), 2021(205 patients) and 2023 (395 patients) were evaluated. Data was inspected visually, and variables that predicted length of stay were included in a Poisson regression model to predict the length of stay.

**Results:**

An analysis of the relationship between year, age, medicolegal issues and length of stay, adjusting for several confounding variables (mobility status, healthcare-acquired infection, long-term care status, need for an increase in homecare package and need for a new homecare package) was carried out as there was an interaction. Charlson’s Comorbidity (CCI) score and delirium were not predictive of length of stay. Medicolegal issues increased length of stay by a factor of 1.46 (95% *CI* 1.39–1.52, *p* < 0.001). For every 1-year increase in age, length of stay increased by a factor of 1.006 in 2019 (95% *CI* 1.004–1.01, *p* < 0.001), 0.988 in 2021 (95% *CI* 0.987–0.99, *p* < 0.001), and 1.004 in 2023 (95% *CI* 1.002–1.01, *p* < 0.001).

**Discussion and conclusion:**

Multimorbidity and delirium did not predict length of stay, while legal issues delayed discharges. On a unit with a baseline longer than average length of stay, there have been progressive improvements in length of stay over time, maybe hastened and retained from lessons learned from the pandemic.

## Introduction

Subacute complex discharge units (CDUs) offer intermediary person-centred care between acute hospital and community services by providing specialised care for patients with complex medical and functional needs [1, 2]. The multidisciplinary team (MDT) model of care that focuses on individual needs enables patients to be discharged to their homes and/or community-based care. Coordination of multidisciplinary services in a Complex Discharge Unit (CDU) is multifaceted and depends on timely collaboration across acute, rehabilitative and community care sectors [3].

Complex hospital discharges normally involve ‘consideration of legal, financial, clinical and practical issues’ [4] and a specialised care plan describing their health and social care needs [5]. The number of complex medical discharges is expected to increase as the population of older adults (≥ 60 years) is expected to increase to 1.4 billion by 2030 and 2.1 billion by 2050 [6]. Likewise, a high proportion of the admissions to acute hospitals comprises older adults with moderate to severe frailty (14.3 to 79.6%) [7], and multimorbidity is highly prevalent (4.7 to 62.8%), depending on the number of targeted conditions [8]. Patients with moderate to severe frailty and those with multimorbidity, i.e. with two or more medical conditions, have various medical and social issues [9].

Several initiatives have been implemented globally to address the challenges of complex discharge planning (CDP). In the UK, the Community Care (Delayed Discharges) Act (CCDA) aims to streamline discharge processes. Canada has introduced the “Alternative Level of Care” designation to categorise patients with complex needs. In the USA, the Better Outcomes for Older People through Safe Transitions (BOOST) initiative focuses on improving care transitions. Australia has adopted a delayed discharge patient placement pathway, supported by an escalation protocol. Meanwhile, Sweden has employed a comprehensive, six-step intervention mapping protocol to address complex discharge issues. These strategies reflect diverse national approaches to managing delayed discharges and improving outcomes for patients with multifaceted care needs [10–14]. In Ireland, the Integrated Care Programme for Older People (ICPOP) supports complex discharge planning by promoting early MDT involvement, identifying frail patients early, and strengthening linkages between hospitals and community providers [15].

Early discharge planning has the potential to/may streamline and potentially shorten the *LOS* of patients admitted to subacute CDUs. These units comprise allied health professionals and a medical team specialising in rehabilitation and medical gerontology to provide care to patients with complex care needs and assist in organising community social support before patients are discharged from the hospital [3].

Several elements of clinical practice were affected during the first year of the COVID-19 pandemic. Interruptions in the regular management of medical conditions contributed to increased complications of comorbid disease, as well as delayed and lost physical follow-ups [16]. The Health Service Executive (HSE) activity in acute public hospitals in Ireland annual report performance published in 2020 reported delayed transfer of care in 11.6% of patients with complex needs; furthermore, 5.2% of patients’ rehabilitation needs were deferred, leading to delays in the transfer of care [16].

Discharge planning exists to reduce the length of stay (*LOS*), as increased *LOS* is associated with worse outcomes and is also an important outcome in measuring the effectiveness of discharge planning [2, 17]. It is expressed in days and is described as the time between admission to and discharge from the hospital [16]. In Ireland, an individual statistical data review on delayed discharge (2016–2018) reported that nearly 90% of older adult patients have delayed discharge but does not define the delayed length of stay days [2, 18]. In Australia and Singapore, delayed discharge is defined as a stay exceeding 21 days and 35 days, respectively [2, 18]. At the same time, in the Netherlands, it is described as a stay that exceeds 50% of the average length of stay for the general population in the previous calendar year [2, 18]. Delayed discharges increase hospital costs, prolong patients’ length of stay and elevate risks such as infections, delirium and ED overcrowding [19]. These could be due to systemic issues like limited bed capacity and delayed transfers, among others. Delayed discharges also contribute to physician burnout by increasing workload and emotional exhaustion, and the impact on provider well-being remains under-researched [19]. As the COVID-19 period was associated with significant disruption to clinical services, assessing subsequent changes in patient outcomes might offer insights for discharge planning.

Our study aimed to determine the impact of several case mix factors on length of stay and how this impact changed across three phases: pre-COVID-19 (2019), during COVID-19 (2021) and late-stage COVID-19 (2023) in a subacute complex discharge unit.

## Methodology

### Ethics

Before collecting data, our institution’s Research and Innovation Office approved the study (reference number: 8900, on the 23rd of May 2024).

### Setting

The CDU context in this study has been described and defined elsewhere [2, 20]. Our CDU is a 23-bed unit in an Irish model 4 academic teaching hospital, referring to a tertiary referral centre, offering a full range of acute medical and surgical specialities. The care team comprises a group of medical doctors (consultant, registrar, senior house officer and an intern), nursing staff and allied healthcare professionals, including physiotherapists, occupational therapists, medical social workers, dieticians and speech and language therapists. The CDU manages medically stable patients who require ongoing medical intervention and/or nursing care and a multidisciplinary team approach to facilitate safe discharges.

### Data source

The data of all patients admitted to our subacute complex discharge unit (CDU) before, during and late-stage of the COVID-19 pandemic were collected. These were defined as patients seen in 2019, 2021 and 2023, respectively. Data were extracted retrospectively from electronic patient records (EPR), as was recorded as part of routine care.

Using an Excel sheet, we extracted the patient’s sex, age, presenting complaint, length of stay, origin of admission, discharge destination, comorbidities and mobility, legal issues and home support needs, among others.

The Charlson Comorbidity Index (CCI) was calculated from the patient’s co-morbid conditions [21]. CCI provides valuable information regarding a patient’s diagnostic and prognostic parameters and predicts mortality [21].

We identified whether patients had medicolegal issues and documented the type of medicolegal issues. These were prompted by the clinical team or family members as part of discharge planning, especially when the question of mental capacity arose. The medicolegal issues we identified were activating enduring power of attorney (EPOA), ward of the court, detention orders and recently decision-making representative (DMR), among others.

According to the Law Society of Ireland, EPOA is “a legal document where you (known as the donor) give another person (known as your Attorney) the power to make decisions and sign legal documents on your behalf” [22]. This is based on the Powers of Attorney Act 1996 [23]. This comes into effect when a person is deemed not to have the capacity to manage their affairs themselves.

A person is made a ward of court by the high court when they cannot manage their assets and/or, in case of a minor (individuals aged less than 18), need protection [24 (22)]. Adult wards of court must be declared medically unfit to manage their assets [24, 25]. In 2015, a new legislation was introduced, the Assisted Decision Making (Capacity) Act, 2015 [26]. This law allows people to make legal agreements on how they can be supported to make decisions about their welfare, property and affairs. This law came into effect in April 2023.

Home and social support services needs were also assessed. To facilitate safe discharge and eliminate and/or reduce readmission, the CDU work to organise social support at home for patients requiring these services. These could be new or augmented forms of support. Home support service is a government initiative aimed at providing support for everyday tasks, such as dressing and undressing, for older people to help them remain in their homes for as long as possible [27].

Furthermore, linking with community teams was also supported, e.g. ICPOP, social clubs and Meals on Wheels, among others. These include government initiatives and voluntary organisations that help older patients remain in their homes for as long as possible. Meals on Wheels is a service that delivers hot meals to the homes of older or disabled people [28].

Acquisition of healthcare-associated infections (HAI) was assessed, and the type of infection was documented. HAIs are described as infection(s) acquired during hospital admission, receiving health care or undergoing a treatment procedure [29]. These have been shown to impact the *LOS* of patients and increase healthcare costs directly [30].

### Statistical analysis

The data was imported into the statistical programming language R [31] and tidied using the Tidyverse package [32]. The Tidyverse was utilised for efficient data management and cleaning, preparing datasets for more in-depth analysis, while also enabling the creation of visually appealing and well-structured visualisations for presentation. The relationships between length of stay and all predictor variables were inspected visually, and variables we felt had predictive power were included in the model. Year was treated as a categorical variable, age as a continuous variable and all other predictors as logical variables.

We used a Poisson regression model to analyse the relationship between year, age, medicolegal issues and length of stay, adjusting for several confounding variables (mobility status, healthcare-acquired infection, long-term care status, need for an increase in homecare package and need for a new homecare package). Year and age appeared to demonstrate an interaction, so we created two models—one with an interaction term and one without. The models were compared, and the model which included the interaction term was chosen as it had superior Akaike Information Criterion (AIC) [33] and Bayesian Information Criterion (BIC) [34] values. The Akaike Information Criterion (AIC) and Bayesian Information Criterion (BIC) are model selection tools that balance goodness of fit and complexity, with AIC favouring predictive accuracy and BIC favouring simpler models, especially as sample size increases. Delirium and CCI were not included in the model, as these did not show a statistically significant relationship with length of stay.

We used the marginal-effects package [35] to calculate the marginal effect of medicolegal status on length of stay and the predicted *LOS* for each combination of year and medicolegal status. To analyse how *LOS* varied by predictors of interest (age, year and medicolegal issues), we standardised for confounding variables by calculating predicted *LOS* for typical patients (defined as having the mean value for continuous predictors, and the modal value for categorical and logical predictors). This typical patient was defined based on the entire cohort rather than for each subgroup. This typical patient was 75.15 years old, was independently mobile, had no healthcare-associated infections, had no long-term care needs, had no need for a new or increased healthcare package and had no medicolegal issues. Predictors of interest were then allowed to vary while confounding variables were held constant. As such, any variation in the predicted outcome was due to the variable of interest and not the confounder. We also used the marginal-effects package [35] to calculate the marginal effect of age on the length of stay for each year.

## Results

### Patient demographics

The characteristics of patients admitted to the subacute CDU are displayed in Table 1.

**Table 1:** The characteristics of patients admitted to the Complex Discharge Unit

	Characteristic	Levels	2019	2021	2023
1	Age, years	*Mean* (*SD*)	79.1 (11.2)	75.2 (13.5)	74.2 (15.1)
2	Gender	*F*, *n* (%)	184 (57.5)	109 (53.2)	197 (49.9)
		*M*, *n* (%)	136 (42.5)	96 (46.8)	198 (50.1)
3	Charlson Comorbidity Index	*Mean* (*SD)*	2.9 (2.5)	1.5 (1.9)	2.3 (2.7)
4	Medicolegal issues	No, *n* (%)	308 (96.2)	199 (97.5)	387 (98.5)
		Yes, *n* (%)	12 (3.8)	5 (2.5)	6 (1.5)
5	Independently mobile	No, *n* (%)	16 (5.0)	11 (5.4)	38 (9.7)
		Yes, *n* (%)	304 (95.0)	193 (94.6)	355 (90.3)
6	Living alone	No, *n* (%)	194 (60.6)	113 (55.4)	219 (55.7)
		Yes, *n* (%)	126 (39.4)	91 (44.6)	174 (44.3)
7	HAI	No, *n* (%)	262 (81.9)	168 (82.4)	172 (43.8)
		Yes, *n* (%)	58 (18.1)	36 (17.6)	221 (56.2)
8	LTC	No, *n* (%)	278 (87.1)	189 (92.6)	364 (92.6)
		Yes, *n* (%)	41 (12.9)	15 (7.4)	29 (7.4)
9	Delirium in CDU	No, *n* (%)	251 (78.4)	183 (89.7)	312 (79.4)
		Yes, *n* (%)	69 (21.6)	21 (10.3)	81 (20.6)
10	Pre-existing HCP	No, *n* (%)	171 (53.4)	148 (72.5)	265 (67.4)
		Yes, *n* (%)	149 (46.6)	56 (27.5)	128 (32.6)
11	Needs increase in HCP	No, *n* (%)	271 (84.7)	177 (86.8)	333 (84.7)
		Yes, *n* (%)	49 (15.3)	27 (13.2)	60 (15.3)
12	Needs new HCP	No, *n* (%)	264 (82.5)	169 (82.8)	331 (84.2)
		Yes, *n* (%)	56 (17.5)	35 (17.2)	62 (15.8)
13	Inpatient death	No, *n* (%)	301 (94.1)	194 (95.1)	385 (98.0)
		Yes, *n* (%)	19 (5.9)	10 (4.9)	8 (2.0)
14	*LOS*, days	*Mean* (*SD*)	51.6 (65.4)	30.7 (34.8)	24.2 (35.3)

Independently mobile refers to mobility status on admission to CDU, and delirium refers to clinically documented episodes during the CDU stay

*SD* standard deviation, *F* female, *M* male, *HAI* healthcare associated# infection, *LTC* long term care, *HCP* Home Care Package, and *LOS* length of stay

### Medicolegal status and year

**Fig. 1:**
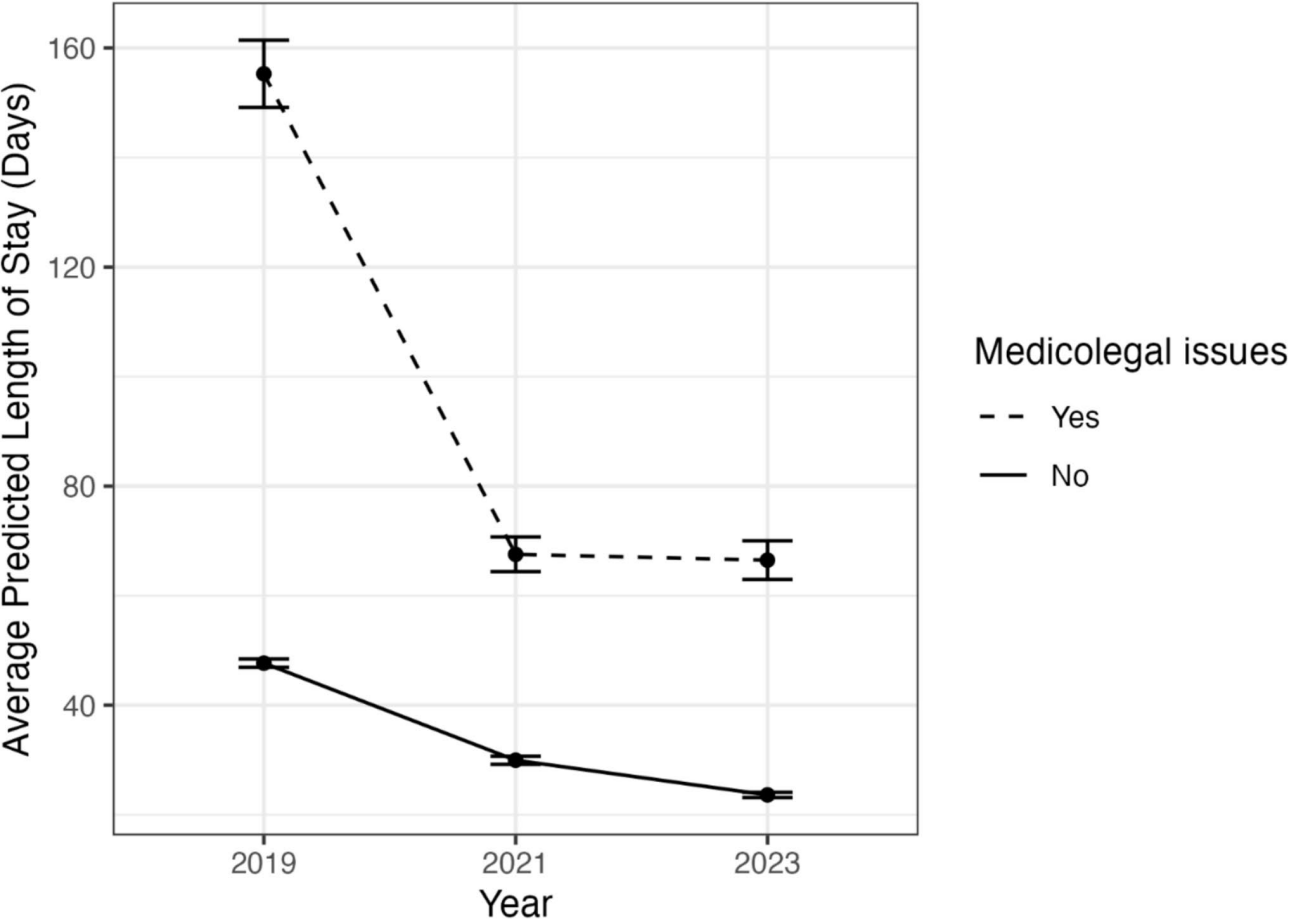
The average predicted *LOS* for patients with and without medicolegal issues

**Fig. 2:**
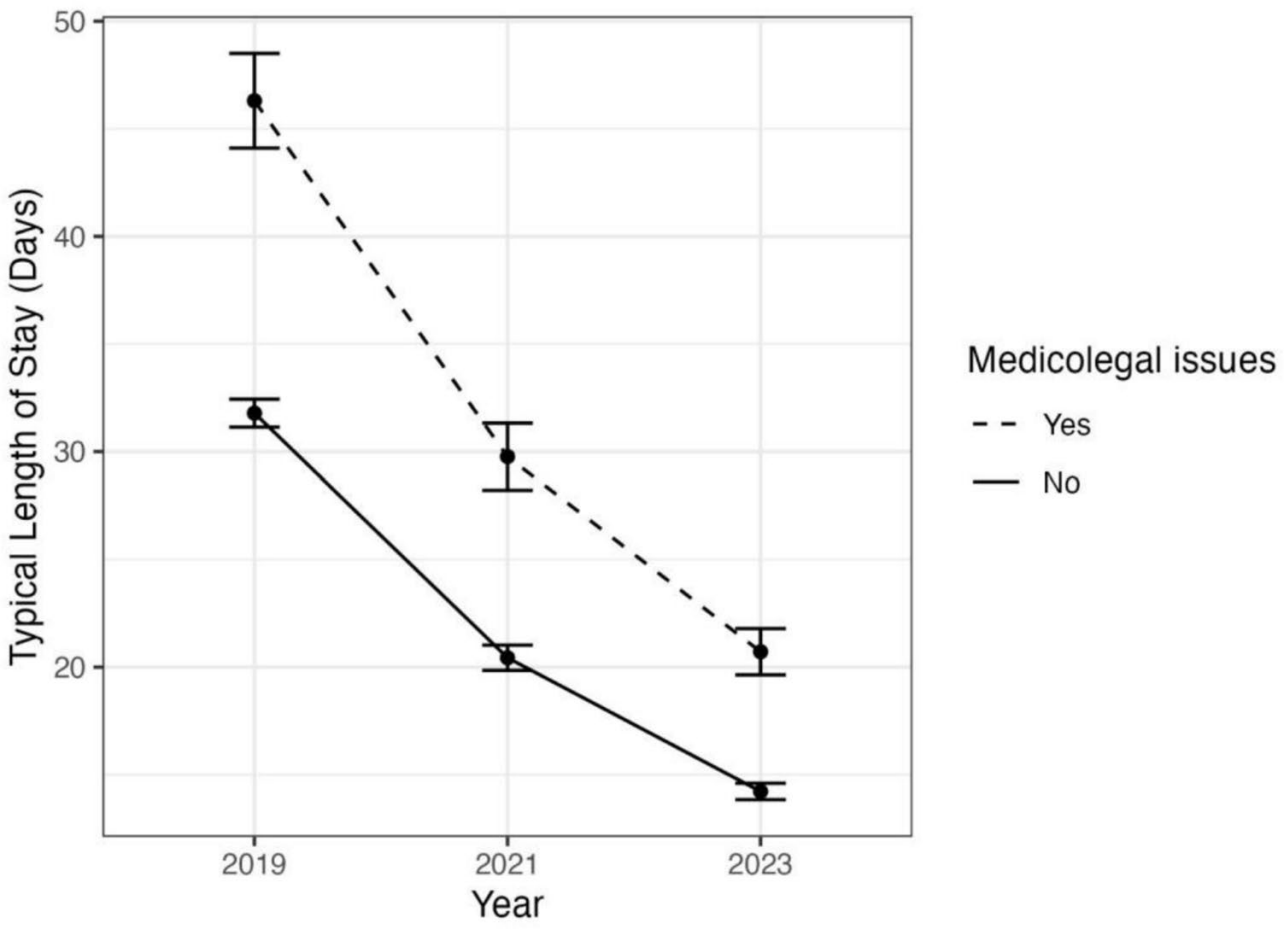
The estimated *LOS* for typical patients with and without medicolegal issues each year

Medicolegal issues increased length of stay by a factor of 1.46 (95% *CI* 1.39–1.52, *p* < 0.001). The average predicted *LOS* for patients with and without medicolegal issues is shown in Fig. 1, and an estimated *LOS* for typical patients with and without medicolegal issues each year is shown in Fig. 2. Table 2 shows the estimated length of stay for typical patients for each combination of year and medicolegal status.

**Table 2:** Estimated length of stay for typical patients for each combination of year and medicolegal status

	Year	Medicolegal issues	Estimated *LOS*, day**s**	95% confidence interval
1	2019	No	31.79	31.14–32.44
2	2021	No	20.43	19.85–21.02
3	2023	No	14.22	13.84–14.6
4	2019	Yes	46.3	44.1–48.5
5	2021	Yes	29.77	28.21–31.31
6	2023	Yes	20.71	19.64–21.79

*LOS* length of stay

### Age and year

**Fig. 3:**
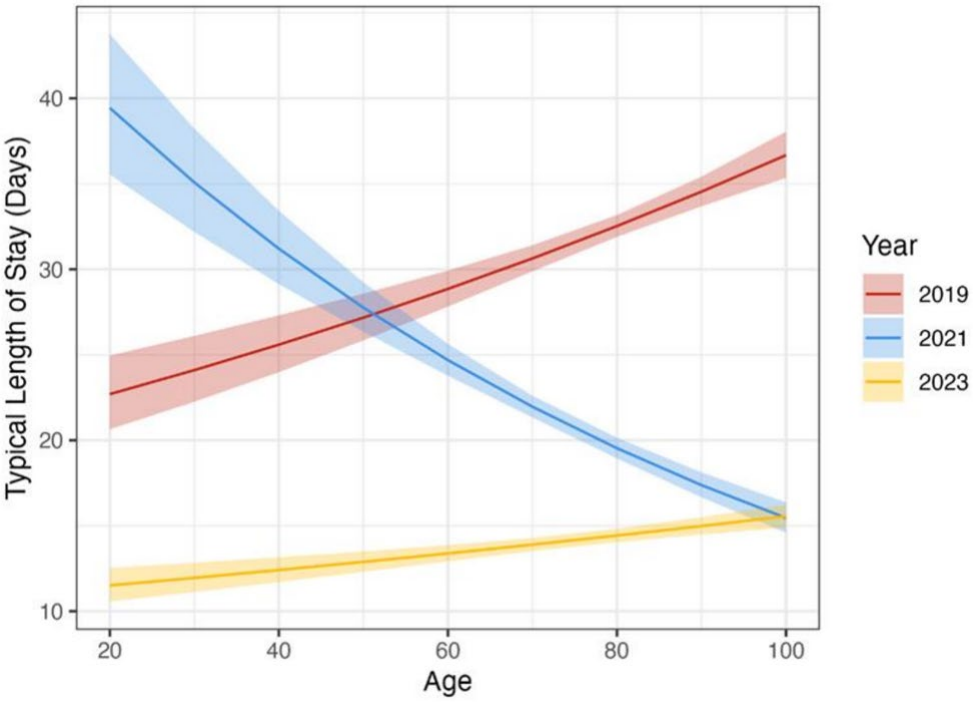
The relationship between *LOS* for a typical patient by age, years and each year. The ribbon shows a 95% confidence interval for the predicted *LOS*

For every one-year increase in age, *LOS* increased by a factor of 1.006 in 2019 (95% *CI* 1.004–1.01, *p* < 0.001), 0.988 in 2021 (95% *CI* 0.987–0.99, *p* < 0.001) and 1.004 in 2023 (95% *CI* 1.002–1.01, *p* < 0.001). While these represent small changes on a year-by-year basis, the cumulative effect is that in 2019, the predicted *LOS* for a typical 40-year-old (defined as having the mean value for continuous predictors and the modal value for categorical predictors) was 25.6 days (95% *CI* 24–27.3), while for a typical 80-year-old, the predicted *LOS* was 32 days (95% *CI* 31.9–33.2). However, this trend reversed in 2021—a typical 40-year-old has a predicted *LOS* of 31.2 (95% *CI* 29.1–33.4), while a typical 80-year-old has a predicted *LOS* of 19.5 (95% *CI* 18.9–20.1). These relationships are shown graphically in Fig. 3.

## Discussion

To our knowledge, this is the first Irish study to evaluate complex discharge patterns across the entire COVID-19 timeline using a subacute CDU lens, comparing the COVID-19 period with the pre-COVID-19 period and the late-stage COVID-19 period. The analysis highlights significant insights into patients’ demographic and clinical characteristics, trends in *LOS* and factors influencing discharge outcomes, particularly when considering the impact of the COVID-19 pandemic.

This study demonstrated variations in patient demographics and characteristics across the observed years. The average age of patients decreased by approximately 5 years over the study period, possibly indicating an adaptation in patients’ admission strategies or shifts in healthcare policies that may have affected the types of patients managed within the CDU. The observed shift toward younger patients in 2023 is likely multifactorial and driven by targeted community supports that enabled older adults to remain at home, high COVID-19-related mortality among the frail (particularly in the Dublin 8 catchment area) [36] and revised triage and referral practices that prioritised complexity over age. Dempsey and colleagues in 2020 revealed that as compared to the rest of the Dublin postal areas, the inner city Dublin and Dublin 8 postal areas (our catchment area) had a higher percentage of the excess COVID-19-related deaths versus deprivation, population density, and proportion of nursing homes within the population relative to pre-COVID-19 period (2015–2019) [36]. A study in England revealed that those aged 80 years or older were seventy times more likely to die with a decremental effect, as compared to those under 40 years [37]. Moreover, it is well documented that the COVID-19 pandemic caused significant limitations in mobility and social isolation, especially in the older population [38]. Concurrently, the maturity of ICPOP initiatives and expanded access to healthcare professionals reduced reliance on CDU admissions for the oldest-old. During the peak of the pandemic, the length of stay for older adults shortened due to emergency discharge protocols, heightened bed capacity pressures and a systemic shift away from admitting highly frail individuals to CDUs, in part due to increased mortality risk.

Overall, there was no significant shift between years in either the demand for increased support or the initiation of new HCPs, suggesting consistency in home care needs across the study period. This suggests that community and discharge planning frameworks, like those embedded through ICPOP and related integrated care models, were robust to demographic shifts and evolving referral practices. The consistency may also reflect unchanged HCP allocation criteria and a potential maximum effect in service provision. Notably, the younger age profile observed in 2023 did not translate into a reduced need for home supports, implying either that complexity and social care needs persist across age bands or that triage processes increasingly prioritised patients with higher support requirements regardless of age.

The presence of medicolegal issues was associated with a marked increase in *LOS*, with patients experiencing a significant delay, compared to those without such issues. This aligns with existing literature, suggesting that medicolegal complexities often result in prolonged hospital stays due to delays in decision-making and coordination required for safe discharge [18]. This critical area is where medicolegal issues can emerge, leading to extended patient stays. The introduction of the Assisted Decision-Making Act in 2023 [26] and the gradual shift from wardship [24] to DMR [39] may have contributed to the notable reduction in medicolegal issues, as seen in the late-stage pandemic (2023). Actively addressing potential issues regarding mental capacity and appointing a DMR in advance may be related to the significant reduction in medicolegal cases, as seen in 2023. This, in turn, would have caused a reduction in the overall *LOS* that can be observed but still higher than those without medicolegal issues. Conversely, in England and Wales, hospital discharge planning for individuals who lack capacity must follow the Mental Capacity Act 2005 and related legislation, which requires best interest decisions and interagency collaboration [40]. Delayed discharges often result from late or disputed capacity assessments, institutional risk aversion, or family conflict, highlighting the need for early assessments, trial discharges and safeguarding of patient autonomy [41].

The COVID-19 period (2021) showed a notable reduction in *LOS*, compared to pre-COVID-19 (2019), which might be attributed to emergency discharge protocols and adaptations in care delivery aimed at reducing hospital burdens [42]. The reduction in medicolegal cases may have contributed to the decreased *LOS* observed in 2023, as medicolegal issues are related to *LOS* [18]. The implementation of daily morning huddles and weekly multidisciplinary team (MDT) meetings led by the consultant geriatrician are in operation mainly during the late-stage COVID-19 period. Furthermore, multidisciplinary rehabilitation is likely related to improved functional outcomes on discharge of older adults with COVID-19 [43]. These initiatives streamlined the discharge process, addressing potential barriers to discharge and mitigating medicolegal concerns, all helping to reduce *LOS*.

Lastly, the delirium and Charlson Comorbidity Index (CCI) did not show a statistically significant relationship with *LOS* in our subacute CDU. This may reflect improved environmental management in CDU, increased awareness of MDT and structured pathways that mitigate delirium-related delays, compared to acute hospital settings. Similarly, a study in Korea of adults aged over 19 years in a rehabilitation unit found that post-transfer days did not show significant differences between patients with delirium and those who did not, as opposed to increased pre-transfer and hospital stay *LOS* [44]. Previous studies have shown increased *LOS* in patients with delirium but were in the acute hospital setting, not in the CDU. Dziegielewski and colleagues in 2021 revealed that delirium increased *LOS* in the ICU and acute hospital stay by approximately a week [45]. Furthermore, a study of older adults aged 65 years or older in the USA revealed that those with delirium had higher *LOS* and increased mortality [46]. The studies on CCI and *LOS* have varied results, with those showing no association, for example, in acute stroke [47] and heart failure [48] and those showing association, mainly surgical studies [49, 50] and acute chest pains [51].

This study makes a novel contribution by examining changes in case mix and length of stay across three phases of COVID-19 in a Complex Discharge Unit. This area has been rarely explored in the existing literature. The findings demonstrate how multidisciplinary interventions and system-level restructuring, such as regular MDT meetings and updated discharge protocols, contributed to improved patient flow. Clinically, this highlights the importance of structured team-based planning in complex discharge management, while also offering a model for future research evaluating intermediate care systems during times of disruption.

## Strengths and weaknesses

A key strength of this study is its repeated cross-sectional design, which allowed for systematic comparisons of patient characteristics, service utilisation and length of stay at distinct time points before, during and in the late stages of the COVID-19 pandemic. This design enabled the identification of temporal trends and the evaluation of healthcare system adaptations over time, without requiring patient-level follow-up. Furthermore, we standardised for confounding variables by calculating predicted *LOS* for typical patients. By the nature of the study being a retrospective review, there may be poorly recorded or incomplete documentation. However, this is reduced by using the electronic patient record system, allowing for easy traceability and transparency. Furthermore, our study was conducted in a single centre, limiting generalisation to other units as the data may differ across units.

## Conclusion

This study highlights the central role of the multidisciplinary team (MDT) in managing complex discharges, particularly in subacute care settings. The reduction in length of stay observed in 2023 aligned with the implementation of weekly MDT meetings, daily morning huddles and a structured late-stage COVID-19 discharge protocol, underscoring the value of regular interdisciplinary collaboration in streamlining patient flow. These structured forums facilitated the earlier identification of barriers to discharge, improved care coordination, enhanced communication across disciplines, enhanced coordination with community services and led to proactive management of medico-legal issues.

The disparities observed in *LOS* across different periods highlight the importance of adaptability in healthcare systems to address emerging challenges and optimise patient flow. Future policy changes, such as fully implementing the Assisted Decision-Making (Capacity) Act and not waiting for acute admissions to deal with medicolegal issues, may alleviate some of the delays currently impacting *LOS*.

## Data Availability

Anonymiosed data supporting the findings of this study are avaliable upon reasonable request.Access to the data requires priorwritten approval from our institution.

## Declarations

I confirm all relevant ethical guidelines have been followed, and ethics approval has been obtained.

### AI use declarations

The author confirms that no AI or AI -assisted technologies were used in the writing process.
